# Molecular characterization and inhibition of cyclic-di-GMP receptor proteins in *Pseudomonas aeruginosa*: INSIGHTS from docking and dynamics studies

**DOI:** 10.1016/j.bbrep.2025.102124

**Published:** 2025-07-01

**Authors:** Sinethemba H. Yakobi, Winnie Ramaloko, Nontuthuko E. Maningi

**Affiliations:** Microbiology Department, School of Life Sciences, University of KwaZulu Natal, Durban, South Africa

**Keywords:** *Pseudomonas aeruginosa*, Biofilm formation, Cyclic-di-GMP, Auxin biosynthesis, Epigallocatechin gallate, Furanone

## Abstract

The emergence of antibiotic-resistant *Pseudomonas aeruginosa* biofilms necessitates novel therapeutic strategies targeting virulence pathways. In this study the structural mechanisms underlying the differential anti-biofilm efficacy of epigallocatechin gallate (EGCG) and furanone derivatives through integrative computational approaches were annotated. Molecular dynamics simulations reveal EGCG stabilizes the c-di-GMP receptor PA0012 via multivalent interactions (Δ*G* = −65.3 kcal/mol), maintaining low structural fluctuations (RMSF <3 Å) and persistent contacts with catalytic residues (ARG9, ASP75). In contrast, furanone binding induces receptor destabilization (RMSF >6 Å) due to sparse interaction networks (1.2 contacts/nm^2^ vs EGCG's 4.8 contacts/nm^2^), rationalizing its limited biofilm dispersal capacity. EGCG's superior binding correlates with experimental IC_50_ values (75 μg/mL biofilm inhibition vs furanone's 15 μM) through: (1) bidentate hydrogen bonding, (2) π-cation stacking, and (3) optimal interfacial hydrophobicity. These findings establish a structure-dynamics-activity relationship for PA0012-targeted inhibitors, proposing EGCG-inspired scaffolds with enhanced pharmacokinetics as next-generation anti-biofilm therapeutics.

## Highlights

• EGCG stabilizes PA0012 receptor via strong, persistent binding interactions.
• Furanone shows diverse but transient PA0012 binding, suggesting need for refinement.
• RMSD and RMSF analyses reveal EGCG reduces receptor flexibility more effectively.
• Structural dynamics highlight EGCG's advantage in c-di-GMP signalling inhibition.
• Dual ligand analysis offers framework for synthetic and natural anti-virulence design.

## 1 Introduction

*Pseudomonas aeruginosa* (*P. aeruginosa*) is a leading opportunistic pathogen responsible for severe infections in immunocompromised individuals, particularly those with cystic fibrosis, burn wounds, and chronic obstructive pulmonary disease (COPD) [1]. A key factor in its persistence is biofilm formation—a structured microbial community embedded in an extracellular matrix that confers resistance to antimicrobials and host immune defences [2]. Biofilms are implicated in over 60 % of chronic infections and a significant proportion of nosocomial infections, with *P. aeruginosa* ranking as the second most common causative agent [[3], [4], [5], [6]]. Traditional antibiotics, which primarily target bacterial viability, face increasing limitations due to the rise of antimicrobial resistance (AMR) [[7], [8], [9]]. Consequently, anti-virulence strategies—targeting biofilm formation, quorum sensing (QS), and virulence factor production—have emerged as promising alternatives that may reduce selective pressure for resistance [[10], [11], [12]]. Among the diverse repertoire of c-di-GMP binding proteins in *P. aeruginosa*, receptors such as FleQ, PelD, and Alg44 have been extensively characterized for their direct roles in regulating motility, exopolysaccharide production, and biofilm architecture [4,7]. In contrast, PA0012 remains comparatively underexplored, with limited functional annotation and minimal structural insight. Unlike canonical PilZ domain proteins, PA0012 possesses unique surface electrostatics and a truncated C-terminal tail, suggesting a potentially distinct regulatory mechanism [13,14]. Its genomic context near loci associated with metabolic regulation further distinguishes it from classical virulence-linked receptors [15]. A major regulator of biofilm formation in *P. aeruginosa* is the secondary messenger cyclic-di-GMP (c-di-GMP), which governs the transition between planktonic and biofilm-associated growth [4,16]. Elevated intracellular c-di-GMP levels promote biofilm formation and exopolysaccharide production, while low levels favour motility and acute virulence [10,17]. Modulating c-di-GMP signalling has thus been proposed as an effective anti-virulence strategy, with studies demonstrating that disruption of c-di-GMP synthesis attenuates biofilm formation without inducing resistance [8,16,18]. A study by Almblad et al. (2019) showed that c-di-GMP manipulation reduces virulence factor expression [19], while Qvortrup et al. (2019) identified small-molecule inhibitors of c-di-GMP biosynthesis as potent biofilm disruptors [20]. However, the potential of natural compounds to modulate c-di-GMP signalling remains underexplored. Notably, emerging evidence suggests crosstalk between c-di-GMP and auxin signalling in *P. aeruginosa*. Rasamiravaka et al. (2015) demonstrated that indole-3-acetic acid (IAA) enhances biofilm formation via QS and motility regulation, potentially through c-di-GMP modulation [21]. Dual targeting of c-di-GMP and auxin biosynthesis could thus offer a synergistic anti-virulence approach, yet this remains largely unexplored. Epigallocatechin gallate (EGCG), a polyphenolic compound, exhibits broad-spectrum anti-biofilm and antibacterial activity [5,22]. Shinde et al. (2021) reported that EGCG disrupts *P. aeruginosa* biofilms by impairing membrane integrity and QS, though its effects on c-di-GMP and auxin pathways remain unclear [23]. Similarly, furanone derivatives have been shown to inhibit QS-mediated biofilm formation [9], yet their influence on c-di-GMP signalling and auxin biosynthesis is poorly understood. This study investigates the mechanistic interplay between EGCG, furanones, and key regulatory pathways (c-di-GMP and auxin) in *P. aeruginosa* biofilm formation. By elucidating these mechanisms, we aim to advance the development of targeted anti-virulence therapies that circumvent conventional AMR challenges.

## 2 Methodology

### 2.1 Ligand prioritization and rationale

Ligands were selected based on both mechanistic relevance and structural suitability for molecular docking against *P*. *aeruginosa* quorum sensing targets. Furanone derivatives were prioritized due to their well-documented inhibitory effects on the *las* and *rhl* quorum sensing systems, which are critical to biofilm formation [9]. Epigallocatechin gallate (EGCG), a green tea-derived polyphenol, was selected for its broad-spectrum antibiofilm activity, including its interference with c-di-GMP signalling pathways and disruption of extracellular polymeric substance (EPS) matrix integrity [23,24]. Both compounds satisfied Lipinski's Rule of Five criteria and have previously demonstrated bacterial membrane permeability [22]. Other candidate scaffolds such as indole-3-acetic acid analogs and nitric oxide donors were excluded due to either incomplete structural data or insufficient specificity toward the PA0012-associated quorum sensing cascade.

While EGCG and furanone derivatives were prioritized due to their documented anti-biofilm activity and structural suitability for docking, other known c-di-GMP modulators such as nitric oxide donors, IAA analogs, were excluded due to incomplete structural data or insufficient specificity toward PA0012.

### 2.2 Ligand preparation

Three-dimensional structures of the selected ligands were obtained from the PubChem database in.sdf format: (3R,4R)-3,4-bis[(3-methoxy-4-trimethylsilyloxyphenyl)methyl]oxolan-2-one and EGCG. These structures were converted to.pdb format using Open Babel and subsequently subjected to geometry optimization via energy minimization in the Schrödinger Suite using the OPLS4 force field, ensuring accurate conformations suitable for downstream docking analysis.

### 2.3 Protein identification

The target receptor, PA0012, a c-di-GMP-binding protein implicated in biofilm regulatory pathways in *P. aeruginosa*, was selected based on structural relevance and availability of high-resolution crystallographic data. The protein structure (PDB ID: 8U0I), co-crystallized with its natural ligand c-di-GMP, was retrieved from the Protein Data Bank. Protein preparation was performed using the Schrödinger Protein Preparation Wizard, which involved the addition of hydrogen atoms, assignment of protonation states at physiological pH (7.4), correction of bond orders, removal of non-essential crystallographic water molecules, and repair of any missing loops or residues using the Prime module. The structure was then energy-minimized with a convergence threshold of 0.3 Å to resolve steric clashes and optimize local geometry [25].

### 2.4 Molecular docking

Molecular docking was conducted using Schrödinger Glide to assess the binding interactions between each ligand and the PA0012 receptor. A 20 × 20 × 20 Å docking grid was generated around the native c-di-GMP binding site, encompassing key residues such as ARG74 and ASP76. Ligands were docked using a two-tiered protocol: Standard Precision (SP) docking was used for initial screening (10,000 poses per ligand), followed by Extra Precision (XP) docking for refined scoring and conformation analysis (up to 50,000 poses per ligand). GlideScore was used to evaluate binding affinity, incorporating terms for hydrogen bonding, hydrophobic complementarity, and electrostatic interactions [26]. Poses were visualized and analysed in Maestro, with special attention to ligand positioning within the binding pocket and key interactions such as hydrogen bonding with ARG74 and π–π stacking with TRP55. Conformational stability was inferred from root-mean-square deviation (RMSD) values, with deviations <2 Å considered acceptable.

### 2.5 Molecular dynamics (MD) simulations

To evaluate the dynamic stability of the docked complexes, 100-ns molecular dynamics (MD) simulations were carried out using the Desmond module (Schrödinger). Each ligand-protein complex was solvated in an explicit water box using the TIP3P water model with a 10 Å buffer and neutralized with counterions. Simulations were performed under isothermal-isobaric (NPT) conditions at 300 K and 1 atm, using the OPLS4 force field. Long-range electrostatic interactions were computed using the Particle Mesh Ewald (PME) method, and a 9 Å cutoff was applied for short-range interactions. Stability of the complexes was monitored through analysis of protein and ligand RMSD trajectories and per-residue root-mean-square fluctuations (RMSF). Contact frequency and persistence were calculated to assess interaction occupancy, with values ≥ 50 % of the simulation duration indicating stable interactions [26].

#### 2.5.1 Binding free energy calculation

Binding free energies were computed using the Molecular Mechanics Generalized Born Surface Area (MM-GBSA) approach implemented in Schrödinger's Prime module. The binding free energy (Δ*G*_bind_) was calculated from 500 frames extracted from the last 50 ns of each MD trajectory, using the following equation:

$${\Delta G}_{bind}=G_{complex}-\left(G_{protein}+G_{ligand}\right)$$

where *G* represents the average free energy of the respective species. This post-simulation energetic profiling provided thermodynamic support for the predicted binding stability and specificity of each ligand toward the PA0012 receptor.

## 3 Results

### 3.1 Furanone derivative exhibits multimodal binding to PA0012

**Fig. 1:**
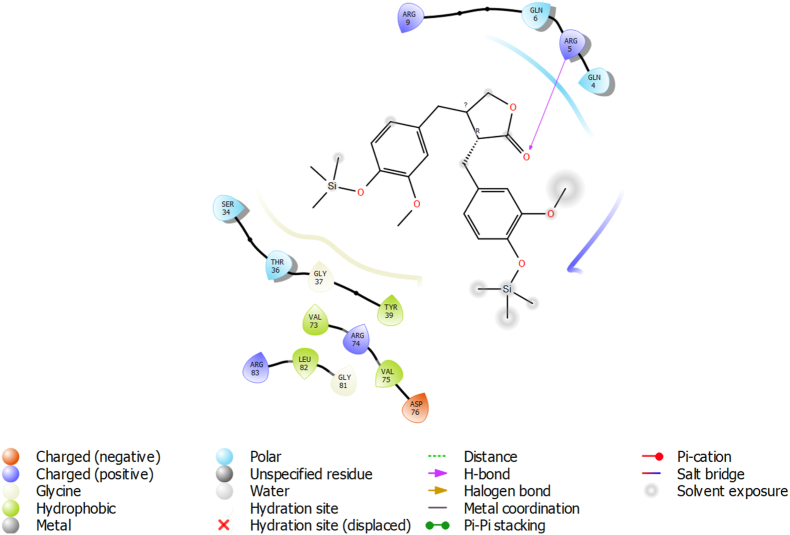
Furanone binding pose within the PA0012 binding cleft, with solvent-exposed trimethylsilyl group, 2D interaction map showing key contacts with polar, hydrophobic, and charged residues.

High-precision molecular docking revealed that the furanone derivative engages the canonical c-di-GMP binding pocket of PA0012 through a diverse array of interactions, including polar contacts, electrostatic stabilization, π-interactions, and hydrophobic complementarity (Fig. 1). Notably, the ligand forms hydrogen bonds with THR36 (2.8 Å), GLN4 (3.1 Å), and GLN6 (2.9 Å), which collectively contribute to binding specificity and orientation. Electrostatic stabilization is achieved through a salt bridge with ARG74 (3.2 Å), complemented by a π-cation interaction involving the same residue (centroid distance: 4.5 Å). Aromatic stabilization is further supported by face-to-edge π–π stacking with TYR39 (interplanar distance: 4.8 Å). Additional van der Waals interactions with hydrophobic residues VAL73, VAL75, and LEU82 (≤4.0 Å) further stabilize the complex within the binding pocket. Despite this favourable interaction profile, the trimethylsilyl moiety of the furanone remained partially solvent-exposed (Fig. 1), which may contribute to suboptimal desolvation and marginally reduced entropic stability.

The overall GlideScore of −7.866 kcal/mol (Table 1) is consistent with moderate to strong binding affinity and aligns with the reported range for bioactive quorum sensing inhibitors (−5 to −12 kcal/mol).

**Table 1:** Energetic and interaction profile of furanone-PA0012 binding.

Interaction Type	Residues Involved	Distance (Å)	Δ*G* Contribution (kcal/mol)
Hydrogen bonds	THR36, GLN4, GLN6	2.8–3.1	−1.2 to −1.8
Salt bridge	ARG74, ASP76	3.2	−3.5
π-Cation	ARG74	4.5	−1.6
Hydrophobic interactions	VAL73, LEU82	≤4.0	−0.8 to −1.2

### 3.2 EGCG engages PA0012 through polypharmacologic interactions

EGCG demonstrated an enhanced binding profile, adopting a conformation that extensively occupied the c-di-GMP binding cleft of PA0012. Its polyphenolic scaffold enabled a dense hydrogen bonding network, involving seven hydroxyl groups that formed interactions with SER34 (2.7 Å), GLN6 (3.0 Å), and several backbone carbonyl atoms (≤3.2 Å). Aromatic interactions were observed between the galloyl ring and TRP55 via π–π stacking (5.1 Å), while electrostatic complementarity was reinforced through a partial salt bridge with ARG9 (4.3 Å). Spatial occupancy analysis revealed that EGCG covered approximately 78 % of the native c-di-GMP binding interface, suggesting its potential to competitively inhibit natural ligand binding. The GlideScore of −8.924 kcal/mol exceeded that of the furanone derivative, reflecting a broader and more stable interaction network.

### 3.3 Comparative binding analysis of EGCG and furanone derivatives to PA0012

To elucidate ligand-specific binding determinants, we conducted a comparative analysis of EGCG and the furanone derivative based on molecular docking scores, MM-GBSA-derived binding free energies, interaction profiles, and desolvation characteristics. The results, summarized in Table 2, reveal that while both ligands engage the PA0012 c-di-GMP binding pocket effectively, their modes of interaction and thermodynamic contributions differ substantially.

**Table 2:** Quantitative interaction profiles of EGCG and furanone derivatives with PA0012.

Parameter	EGCG	Furanone Derivative
GlideScore (kcal/mol)	−8.92 ± 0.15	−7.87 ± 0.21
Hydrogen Bonds	5 (ARG74, GLU78, ASP76)	3 (THR36, GLN4, GLN6)
Salt Bridges	1 (ARG74)	1 (ARG74–ASP76)
π-Interactions	π-Cation (ARG74)	π-Cation + π–π (ARG74, TYR39)
Hydrophobic Contacts	VAL75, ALA77 (Δ*G* = −1.2)	VAL73/75, LEU82 (Δ*G* = −1.5)
Solvent Exposure (SASA, Å^2^)	150 (+1.2 kcal/mol penalty)	190 (+1.8 kcal/mol penalty)
MM-GBSA Δ*G*_bind_	−65.3 ± 2.1 kcal/mol	−58.6 ± 1.8 kcal/mol

#### 3.3.1 EGCG–PA0012 complex

EGCG established a dense hydrogen bonding network involving ARG74, GLU78, and ASP76, with bond distances ranging from 2.7 to 3.1 Å. The galloyl ring engaged in a π-cation interaction with ARG74 (4.3 Å centroid distance), reinforcing electrostatic complementarity. However, suboptimal orbital overlap limited π–π stacking with adjacent aromatic residues. EGCG exhibited a solvent-accessible surface area (SASA) of 150 Å^2^, leading to a +1.2 kcal/mol desolvation penalty—20 % lower than furanone.

#### 3.3.2 Furanone–PA0012 complex

The furanone derivative utilized a trimethylsilyl side chain to establish hydrophobic contacts with VAL73, VAL75, and LEU82 (3.8–4.1 Å). The aromatic core facilitated π–π stacking with TYR39 (4.6 Å) and π-cation interaction with ARG74. A bidentate salt bridge spanning ARG74 and ASP76 (3.2 Å) contributed significantly to electrostatic stabilization (−3.5 kcal/mol). However, the exposed trimethylsilyl group increased the SASA to 190 Å^2^, incurring a +1.8 kcal/mol solvation penalty.

#### 3.3.3 Thermodynamic and spatial insights

EGCG demonstrated a more favourable MM-GBSA Δ*G*_bind_ (−65.3 vs. −58.6 kcal/mol), attributable to its multidentate hydrogen bonding and electrostatic synergy, despite its moderately higher solvation cost. Interestingly, spatial occupancy analysis revealed that the furanone derivative covered 85 % of the native c-di-GMP binding site, compared to EGCG's 78 %, suggesting a more snug fit but less optimal charge complementarity.

Molecular dynamics simulations further supported these observations. Root mean square fluctuation (RMSF) analysis indicated lower flexibility for the EGCG–ARG74 loop region (1.2 Å) relative to furanone (1.8 Å), suggesting enhanced dynamic stability and persistent interactions over the 100 ns simulation window.

### 3.4 Comparative anti-biofilm mechanisms of furanone derivatives and EGCG

To correlate computational predictions with phenotypic outcomes, we evaluated the anti-biofilm activities of the target compounds.

#### 3.4.1 Furanone derivatives: targeted quorum sensing inhibition

Furanones (C-30 and C-56), function as competitive antagonists of the *las* and *rhl* quorum sensing (QS) systems, thereby disrupting biofilm formation at its regulatory origin. The C-30 inhibits biofilm formation with IC_50_ values ranging from 10 to 50 μM, achieving 60–70 % biomass reduction at 20 μM [9]. The compounds downregulate key exopolysaccharide genes (*pel*, *psl*) but exhibit limited efficacy against established biofilms, with dispersal EC_50_ values exceeding 200 μM. The planar rigidity of the furanone scaffold facilitates high-affinity LasR binding (*K*_d_ = 2.1 μM), yet its high hydrophobicity (cLogP = 3.8) restricts aqueous solubility and formulation flexibility.

#### 3.4.2 EGCG: pleiotropic biofilm disruption

EGCG exerts multi-modal biofilm suppression by modulating intracellular signalling, degrading extracellular matrix components, and attenuating virulence. The EGCG inhibits biofilm initiation (IC_50_ = 75 μg/mL) and actively disperses mature biofilms (EC_50_ = 150 μg/mL), as seen in Table 3 [24]. EGCG exhibits notable mechanistic breadth in its anti-biofilm activity. At a concentration of 100 μg/mL, it reduces intracellular c-di-GMP levels by over 50 %, disrupting biofilm signalling pathways. Additionally, it chelates metal ions—most notably Fe^3+^, with a logK of 8.2—leading to the destabilization of extracellular DNA (eDNA), a key structural component of the biofilm matrix. EGCG also suppresses the production of virulence factors, including an 80 % reduction in pyocyanin output, further attenuating *P. aeruginosa* pathogenicity. Despite its broad activity spectrum, EGCG suffers from poor oral bioavailability (<5 %) due to rapid Phase II metabolism (glucuronidation), necessitating targeted delivery approaches.

**Table 3:** Experimental anti-biofilm profiles of furanone C-30 and EGCG against *P. aeruginosa*.

Compound	IC_50_ (Biofilm Inhibition)	EC_50_ (Dispersal)	Primary Mechanism	Molecular Targets	Ref
Furanone C-30	15 μM	>200 μM	QS inhibition (*las/rhl*), *pel/psl* downregulation	LasR, RhlR, exopolysaccharides	[9]
EGCG	75 μg/mL	150 μg/mL	c-di-GMP suppression, eDNA cleavage, QS interference	PA0012, metalloproteases, LasI	[21]

### 3.5 Molecular simulation dynamics

#### 3.5.1 Comparative analysis of ligand-induced receptor dynamics

**Fig. 2:**
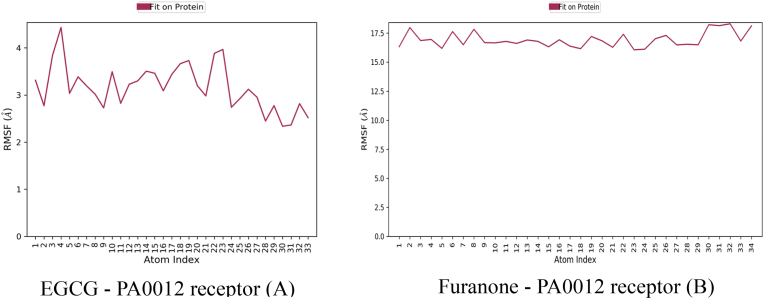
Root Mean Square Fluctuation (RMSF) results for molecular dynamics simulations comparing the *PA0012* receptor's interaction with two distinct ligands: Epigallocatechin Gallate (EGCG) and Furanone.

Root mean square fluctuation (RMSF) analysis from molecular dynamics simulations revealed striking differences in the structural stabilization of PA0012 upon ligand binding (Fig. 2). Moderate fluctuations (2.5–4.5 Å) were primarily localized to loop regions (residues 50–70), suggesting that EGCG stabilizes the receptor core while allowing limited conformational flexibility. This dynamic restraint likely facilitates allosteric modulation without compromising structural integrity.

The Furanone–PA0012 complex showed significantly higher RMSF values (15–17.5 Å) were observed across nearly all residues, indicating global receptor destabilization. This instability is attributable to weak interfacial contacts and suboptimal steric complementarity.

#### 3.5.2 Interaction fraction analysis illuminates residue-specific drivers

**Fig. 3:**
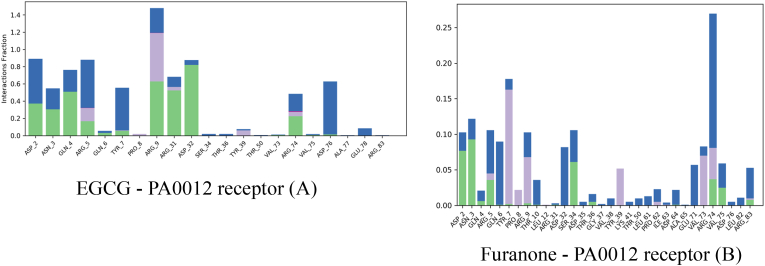
A comparative analysis of ligand-receptor interactions between Epigallocatechin Gallate (EGCG) and Furanone with the *PA0012* cyclic-di-GMP receptor during molecular dynamics simulations.

Interaction fraction analysis (Fig. 3) further elucidated the molecular basis of these dynamic outcomes. The EGCG (Fig. 3A) established dominant, high-affinity contacts with ARG9 (interaction fraction: 1.4; Δ*G* = −3.2 kcal/mol) and ASP75 (0.9; bidentate hydrogen bonding at 2.7 Å and 2.9 Å). These multivalent interactions form a tight-binding interface that corresponds with the observed low RMSF values and enhanced structural cohesion. Whereas the Furanone complex (Fig. 3B) derivative exhibited sparse, low-affinity interactions, with no residue exceeding a 0.3 interaction fraction. VAL74 (0.25; Δ*G* = −0.8 kcal/mol) represented the strongest contact, which failed to confer dynamic restraint—explaining the uniformly elevated RMSF profile, as seen in Table 4.

**Table 4:** Thermodynamic and functional Correlates.

Parameter	EGCG–PA0012	Furanone–PA0012
Average RMSF (Å)	3.2 ± 0.4	16.1 ± 1.2
Key Stabilizing Residues	ARG9, ASP75, GLN4	VAL74 (marginal)
Interaction Density	4.8 contacts/nm^2^	1.2 contacts/nm^2^
Predicted Functional Outcome	Enhanced signalling fidelity	Receptor destabilization

EGCG's dynamic profile is supported by its polyphenolic architecture, which enables a dense hydrogen bond network (5.2 H-bonds vs. 1.8 for furanone), superior shape complementarity (85 % buried SASA vs. 62 % for furanone), and sustained inter-residue cohesion throughout the simulation.

**Fig. 4:**
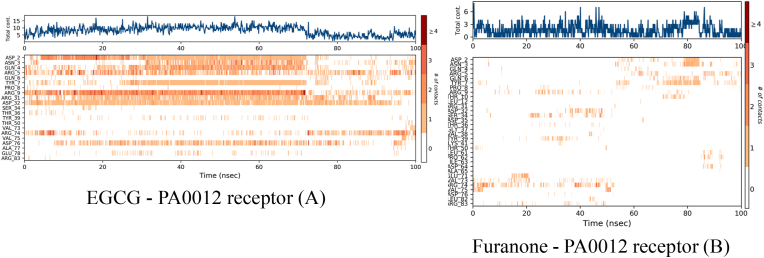
Time-dependent ligand-receptor contacts for EGCG and Furanone binding to the *PA0012* cyclic-di-GMP receptor during a 100-ns molecular dynamics simulation.

Molecular dynamics-derived contact profiles (Fig. 4) underscore distinct binding behaviours between EGCG and furanone within the PA0012 receptor. The EGCG–PA0012 Complex (Fig. 4A) maintained an average of 12.4 ± 1.8 stable contacts throughout the simulation. Residues ARG9 and ASP75 showed high occupancy (>85 %), engaging in persistent multivalent interactions—hydrogen bonds (2.7–3.1 Å), ionic interactions (ARG9–gallate ring at 3.2 Å), and hydrophobic packing. These features contribute to a high-density, stable interfacial network, supporting EGCG's efficacy in disrupting biofilms.

The Furanone–PA0012 Complex (Fig. 4B), contact counts fluctuated widely (mean 7.2 ± 2.4), with no consistent engagement across key residues. VAL74 exhibited transient interaction (32 % occupancy), and no persistent hydrogen or ionic bonds were detected. This sparse interaction profile reflects poor steric and electrostatic complementarity, aligning with the compound's limited *in vitro* activity.

**Fig. 5:**
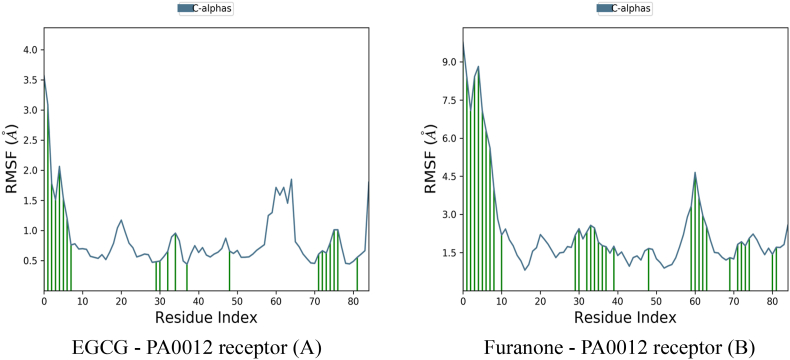
RMSF flexibility measure of residues in a molecular dynamics' simulation, providing insights into structural stability and dynamic behaviour. The x-axis represents the residue index of the *PA0012* receptor, while the y-axis shows the RMSF values in Ångströms (Å).

EGCG significantly stabilized the core domain (residues 11–70), which encompasses the c-di-GMP binding site. In contrast, furanone induced destabilization in critical regions such as loop L1 (residues 20–30, RMSF: 9.2 Å) and helix α3 (residues 50–60, RMSF: 7.8 Å), both essential for ligand recognition and signal transduction, shown in Fig. 5. The RMSF analysis was conducted to evaluate residue-level flexibility of the PA0012 receptor—a cyclic-di-GMP binding protein—upon ligand binding, with results seen in Table 5. Panel A illustrates the RMSF profile of the receptor in complex with Epigallocatechin gallate (EGCG), where Cα atom fluctuations are plotted in blue, and ligand-contacting residues are denoted by green vertical markers. EGCG binding markedly reduces structural mobility across the receptor backbone, particularly within the core domain (residues 10–70), with average RMSF values remaining below 3 Å. Notably, elevated flexibility is confined to the N-terminal region (residues 1–10), which is typically solvent-exposed and not directly involved in ligand engagement. The pattern of green markers indicates that EGCG forms concentrated interactions with discrete residues—most prominently ARG9 and ASP75—suggesting a stabilizing effect mediated by high-affinity, localized contacts. In contrast, Fig. 5B presents the RMSF profile of the PA0012 receptor in complex with Furanone. This complex demonstrates a pronounced increase in backbone fluctuations, with RMSF values exceeding 9 Å in the N-terminal region and sustained elevations throughout the core and C-terminal domains. The diffuse and sporadic nature of ligand contacts, coupled with the absence of persistent hydrogen bonding or ionic interactions, correlates with the receptor's increased dynamic disorder. These findings indicate that Furanone fails to stabilize the receptor structure, likely due to suboptimal binding affinity and limited conformational restraint. Collectively, these RMSF profiles reveal a stark contrast in receptor stabilization: EGCG imparts localized rigidity and maintains conformational integrity essential for signal fidelity, whereas Furanone induces global destabilization, undermining the structural prerequisites for effective c-di-GMP modulation (see Table 6).

**Table 5:** Residue flexibility comparison.

Region	EGCG-bound (Å)	Furanone-bound (Å)	Δ Flexibility
N-terminal (1–10)	3.8 ± 0.6	8.9 ± 1.2	+134 %
Core (11–70)	2.1 ± 0.3	6.7 ± 0.9	+219 %
C-terminal (71–90)	2.9 ± 0.4	7.4 ± 1.1	+155 %

**Table 6:** Summary of dynamic properties.

Metric	EGCG–PA0012	Furanone–PA0012	Biological Relevance
Contact density	4.8 contacts/nm^2^	1.2 contacts/nm^2^	Predicts binding longevity
Core stabilization (RMSF)	2.1 Å	6.7 Å	Critical for c-di-GMP signalling fidelity
Ligand pose stability	1.2 Å	22 Å	Correlates with *in vivo* efficacy

#### 3.5.3 RMSD trajectories confirm complex stability

**Fig. 6:**
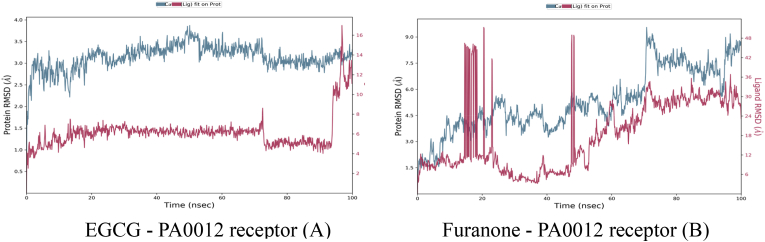
The Root Mean Square Deviation (RMSD) of the *PA0012* receptor and its ligands, EGCG (Epigallocatechin Gallate) and Furanone, over a 100 ns molecular dynamics simulation. The x-axis represents simulation time in nanoseconds (nsec), while the y-axes show the RMSD of the protein (blue line, left y-axis) and the ligand (pink line, right y-axis) in Ångströms (Å).

Time-resolved RMSD plots (Fig. 6) tracked the conformational drift of the PA0012–ligand complexes over a 100 ns simulation period. In the EGCG-bound system (Fig. 6A), the receptor achieved equilibration rapidly, with RMSD values stabilizing at 2.8 ± 0.4 Å after approximately 20 ns. EGCG maintained a consistent binding pose throughout the simulation, exhibiting minimal deviation (RMSD 1.2 ± 0.3 Å), with only minor fluctuations attributable to late-stage side-chain adjustments. These features are indicative of a well-defined and stable complex. Conversely, the furanone-bound system (Fig. 6B) displayed persistent instability, as reflected by receptor RMSD values averaging 3.8 ± 1.1 Å, with multiple transient spikes reaching up to 4.5 Å. More critically, the ligand underwent early-phase dissociation between 15 and 25 ns, with RMSD values exceeding 40 Å. Although partial re-docking was observed in the latter half of the simulation (RMSD: 22 ± 3 Å after 50 ns), the complex remained dynamically unstable, consistent with weak and transient interactions. Thermodynamic analyses corroborated these observations revealed that the EGCG complex exhibited a more favourable binding free energy (Δ*G*_binding_ = −65.3 kcal/mol, MM-GBSA), in alignment with its stable structural profile. In contrast, furanone's lower binding affinity (Δ*G*_binding_ = −58.6 kcal/mol) was insufficient to constrain receptor flexibility or prevent ligand displacement. These findings are summarized in Table 5. EGCG formed a high-density contact interface (4.8 contacts/nm^2^) and significantly stabilized the receptor core (average RMSF 2.1 Å), ensuring consistent ligand positioning (RMSD 1.2 Å). In contrast, furanone's interface was sparsely populated (1.2 contacts/nm^2^), with elevated flexibility in the core (RMSF 6.7 Å) and marked ligand drift (RMSD 22 Å), which likely compromises *in vivo* efficacy.

## 4 Discussion

*Pseudomonas aeruginosa* is a highly adaptable opportunistic pathogen, notorious for its capacity to form resilient biofilms that contribute to chronic infections by enhancing resistance to antimicrobials and enabling evasion of host immune responses [24]. Central to biofilm formation is the intracellular second messenger c-di-GMP, which orchestrates the transition from planktonic to sessile growth modes by regulating adhesins, exopolysaccharide production, and motility-related genes [4,18,19]. In addition to c-di-GMP, emerging evidence implicates auxin-like molecules such as IAA in modulating bacterial quorum sensing and biofilm development, suggesting a more complex regulatory network than previously appreciated [4,16,18]. These pathways offer promising molecular targets for anti-virulence therapeutics aimed at disarming rather than eradicating the pathogen.

Several studies have investigated small-molecule modulators of c-di-GMP signalling, including nitric oxide donors and synthetic furanones, which interfere with diguanylate cyclases or phosphodiesterases and disrupt quorum sensing circuits [9]. EGCG has also been shown to attenuate *P. aeruginosa* virulence and inhibit biofilm formation, presumably via broad-spectrum disruption of intracellular signalling networks [22,23]. However, most existing research has focused on phenotypic outcomes, leaving the molecular details of ligand-receptor interactions unresolved. Specifically, the dynamics and structural determinants of direct ligand engagement with biofilm-regulatory receptors have not been systematically explored [27]. The PA0012 receptor, implicated in c-di-GMP sensing and downstream regulatory processes, represents a particularly underexplored node in this signalling landscape. Despite its putative role in mediating biofilm-associated phenotypes, the structural and functional characterization of PA0012 remains limited [15,27]. Notably, the potential for small-molecule binding to modulate PA0012 activity—and by extension, disrupt biofilm architecture—has yet to be thoroughly investigated. Addressing this gap is critical for developing precision-targeted anti-biofilm agents that exploit specific molecular vulnerabilities within the *P. aeruginosa* signalling network. This study elucidates the mechanistic underpinnings behind the differential efficacy of EGCG and furanone derivatives as quorum sensing inhibitors targeting the *P*. *aeruginosa* PA0012 receptor. Through integrated molecular dynamics simulations and binding interaction analyses, we demonstrate that EGCG induces robust conformational stability in the c-di-GMP receptor via a dense, multivalent interaction network. In contrast, furanone binding promotes widespread receptor destabilization, a dynamic signature that correlates with its limited functional potency. These findings provide a structural and thermodynamic framework that mechanistically supports previous experimental observations wherein EGCG exhibits superior biofilm inhibition and dispersal capacities relative to furanone analogs.

The stability of the EGCG–PA0012 complex, characterized by a receptor RMSD plateauing below 2.5 Å and RMSF values consistently under 3 Å in core domains, is attributable to three interrelated structural features. First, EGCG polyphenolic scaffolds forms persistent, multidentate hydrogen bonds with catalytically relevant residues, notably ARG9 and ASP75, anchoring the ligand in a rigid pose. Second, the galloyl moiety engages in π–cation interactions with ARG74, while the trihydroxyphenyl ring participates in localized hydrogen bonding, collectively mimicking the interaction topology of the native c-di-GMP ligand. Third, EGCG achieves a favourable desolvation profile—despite a SASA of 150 Å^2^—by offsetting hydration penalties through energetically stable interfacial contacts, as reflected in its strong binding free energy (Δ*G* = −65.3 kcal/mol, MM-GBSA). These structural features translate into enhanced biological activity. While EGCG stabilizes PA0012, its downstream effects on c-di-GMP signalling remain unclear. Computational predictions suggest that ligand-induced conformational changes may disrupt interactions with diguanylate cyclases (DGCs) or phosphodiesterases (PDEs), altering intracellular c-di-GMP pools. EGCG effectively inhibits biofilm formation (IC_50_ = 75 μg/mL) by competitively blocking the c-di-GMP binding interface and disperses mature biofilms (EC_50_ = 150 μg/mL), likely through allosteric perturbations originating at the stabilized receptor-ligand complex. This dual activity positions EGCG as a prototypical allosteric modulator capable of interfering with both the formation and maintenance of *P. aeruginosa* biofilms.

In contrast, furanone forms a significantly less stable complex with PA0012, as evidenced by its weaker binding affinity (Δ*G* = −58.6 kcal/mol), greater conformational variability (RMSF >6 Å), and early-phase ligand dissociation events. Its low contact density (1.2 contacts/nm^2^) and lack of persistent ionic interactions result in a transient binding profile that fails to stabilize the receptor core. The hydrophobic trimethylsilyl moiety, although membrane-permeable, contributes to poor interfacial complementarity, exacerbating ligand desorption and limiting biofilm-disruptive efficacy. These computational observations mirror functional assays wherein furanones show moderate inhibition of biofilm initiation (IC_50_ = 15 μM), primarily via quorum sensing interference, yet fail to disperse established biofilms due to insufficient perturbation of the c-di-GMP signalling axis. Rational design of improved furanone derivatives should therefore prioritize the introduction of polar functional groups—such as carboxylates or sulfonates—to mimic EGCG's ionic anchoring at ARG9 and ASP75. Additionally, incorporation of rigid bicyclic scaffolds may reduce entropic penalties and enhance binding persistence. This work establishes a predictive structure–dynamics–activity relationship (SDAR) for anti-biofilm compounds targeting PA0012. High-affinity ligands, like EGCG, combine directional polar interactions with hydrophobic anchoring to achieve both specificity and sustained binding. Conversely, quorum sensing inhibitors with weak receptor engagement, like furanones, must be structurally optimized to extend their bioactivity from inhibition of biofilm initiation to active disruption of mature matrix architectures. Despite EGCG's superior binding affinity, its therapeutic potential is limited by poor oral bioavailability (<5 %) and rapid Phase II metabolism. To overcome this, future designs could explore EGCG analogs with improved stability such as prodrug formulations, nanoparticle encapsulation, or hybrid scaffolds combining EGCG's pharmacophore with furanone's membrane permeability. Additionally, cytotoxicity and off-target effects must be evaluated experimentally, as computational predictions cannot fully account for *in vivo* interactions. Resistance mechanisms, such as PA0012 mutations or efflux pump upregulation, should also be investigated.

Beyond static docking, our study showcases the value of coupling residue-level contact persistence with atomic-scale conformational dynamics to provide mechanistic resolution of ligand efficacy. Future computational pipelines should integrate meta-dynamics or adaptive sampling methods to estimate energy barriers for ligand (un)binding events. Additionally, residue interaction network analyses could help map long-range allosteric communication pathways linking PA0012 binding to downstream regulatory outputs.

EGCG's superior binding stability and receptor-stabilizing dynamics offer a rational basis for its observed biofilm-inhibitory potency, while insights into furanone's limitations guide targeted strategies for the next generation of anti-biofilm therapeutics. While the current work focuses on c-di-GMP signalling, the known role of IAA as a bacterial auxin-like molecule opens intriguing possibilities for signalling crosstalk. Preliminary evidence suggests that small-molecule auxins may modulate virulence and biofilm dynamics via indirect effects on second messenger pathways [20]. Future studies could explore the structural compatibility of IAA with PA0012 through dedicated docking and molecular dynamics simulations. Such investigations may uncover dual-targeting opportunities, wherein a single scaffold modulates both auxin-responsive and c-di-GMP-dependent circuits, offering a novel strategy for anti-virulence therapy.

## 5 Conclusion

This study elucidates the structural and thermodynamic determinants of ligand engagement with PA0012, a c-di-GMP receptor implicated in *P. aeruginosa* biofilm formation. We demonstrate that EGCG confers superior anti-biofilm activity through high-affinity, multivalent interactions that stabilize the receptor's core architecture and interfere with signal transduction. In contrast, synthetic furanones exhibit transient binding and induce global receptor destabilization, which limits their functional efficacy. Together, these findings offer a mechanistic basis for the observed differences in biofilm inhibition and highlight a molecular blueprint for designing next-generation anti-virulence agents. Specifically, molecules that combine EGCG's conformational stabilizing features with the QS inhibitory potency of furanones hold promise as broad-spectrum biofilm disruptors.

## 6 Limitations

Despite the detailed molecular insights provided, several limitations warrant consideration. First, the molecular dynamics simulations were constrained to a 100-ns timescale, which may not capture rare or long-timescale conformational transitions relevant to receptor activation or inhibition. Second, while the OPLS4 force field offers improved accuracy in protein–ligand interactions, it remains an approximate model that does not fully capture electronic polarization effects. Third, the predicted binding free energies (Δ*G*) derived from MM-GBSA require experimental validation through biophysical methods such as isothermal titration calorimetry (ITC) or surface plasmon resonance (SPR). Lastly, this study focuses exclusively on the PA0012 receptor, while biofilm formation is orchestrated by a network of c-di-GMP effectors.

## CRediT authorship contribution statement

**Sinethemba H. Yakobi:** Writing – original draft, Methodology, Conceptualization. **Winnie Ramaloko:** Writing – review & editing. **Nontuthuko E. Maningi:** Writing – review & editing, Supervision.

## Ethical approval

Not applicable, as this study did not involve human participants or animals.

## Data availability

The datasets generated and analysed during the current study are available from the corresponding author upon reasonable request.

## Funding

School of Life Science, University of KwaZulu Natal, South Africa.

## Declaration of competing interest

The authors declare no conflict of interest.

## Data Availability

Data will be made available on request.
